# Merosin-deficient congenital muscular dystrophy type 1A: A case report

**DOI:** 10.3892/etm.2013.1271

**Published:** 2013-08-23

**Authors:** ZHANWEN HE, XIANGYANG LUO, LIYANG LIANG, PINGGAN LI, DONGFANG LI, MENG ZHE

**Affiliations:** Department of Pediatrics, Sun Yat-sen Memorial Hospital of Sun Yat-sen University, Guangzhou, Guangdong 510120, P.R. China

**Keywords:** laminin α-2 gene, molecular genetic testing, merosin-deficient congenital muscular dystrophy type 1A, white matter abnormalities

## Abstract

The aim of this study was to characterize the clinical and genetic features of a 4-year-old female with merosin-deficient congenital muscular dystrophy type 1A (MDC1A). MDC1A is the most common form of congenital muscular dystrophy. MDC1A is caused by mutation of the laminin α-2 gene (LAMA2), localized to chromosome 6q22–23. Clinical presentation, as well as the results of neuro-imaging, electrophysiology and molecular genetic tests were used to evaluate a patient with MDC1A. The patient exhibited severe hypotonia and marked proximal weakness at 6 months of age, as well as delayed developmental milestones. The serum creatine kinase levels of the patient were elevated at 1,556 IU/l. Magnetic resonance imaging (MRI) showed that the white matter in the frontal, parietal, temporal and occipital lobes was abnormal with low signal intensities on T1-weighted images and high signal intensities on T2-weighted images; however, the cortex was normal. Sequencing of the 65 exons of the LAMA2 revealed a homozygous nonsense mutation in exon 50: a C>T exchange in nucleotide 7147 that resulted in a stop codon (Arg2383X stop). Molecular genetic testing is a reliable method for confirming a diagnosis of MDC1A. When a patient presents with severe congenital hypotonia, muscle weakness, high serum creatine kinase (CK) levels and white matter abnormalities, the evaluation may directly proceed to molecular genetic testing of the LAMA2 gene without performing a muscle biopsy.

## Introduction

Congenital muscular dystrophies (CMDs) are genetically and clinically heterogeneous hereditary myopathies that have a predominantly autosomal recessive mode of inheritance. They are characterized by congenital hypotonia, delayed motor development and the early onset of progressive muscle weakness, as well as a dystrophic pattern on muscle biopsy (1). Merosin-deficient congenital muscular dystrophy type 1A (MDC1A) is one of the most frequent forms of CMD in Western countries. It is an autosomal recessive neuromuscular disorder caused by mutations in the laminin α-2 gene (LAMA2) on chromosome 6q22–23 that results in a deficiency of the laminin α-2 chain, a component of skeletal muscle extracellular matrix laminin-2, merosin (2).

In China, MDC1A is an extremely rare condition with only seven reported cases to date (3). Patients with MDC1A have severe muscular weakness and atrophy, diffuse contractures, inability to walk and facial dysmorphism. In addition, they have markedly increased serum creatine kinase (CK) levels and have characteristic white matter abnormalities on cranial magnetic resonance imaging (MRI).

With more widespread use of molecular genetic testing, these tests are becoming more important for confirming the diagnosis of CMD subtypes than are muscle biopsies. In the current study, we report a case of a young female with MDC1A whose diagnosis was confirmed by clinical presentation, characteristic white matter abnormalities and molecular genetic testing without the need for a muscle biopsy.

## Case report

### Clinical presentation

The 4-year-old patient was the first daughter of non-consanguineous, healthy parents. The patient first attended the Department of Pediatrics, Sun Yat-sen Memorial Hospital of Sun Yat-sen University (Guangdong, China) at the age of 3 years due to severe hypotonia and marked proximal weakness. Developmental milestones were delayed at 6 months of age and the patient exhibited severe axial and peripheral hypotonia with feeding difficulties. By 2 years of age, the patient was able to hold her head up, but was unable to roll over or sit alone. At the age of 4 years, the patient was able to sit unsupported, but not stand. Intellectual and speech development was normal. The individual was born at 41 weeks of gestation and the birth weight was 3,100 g. The pregnancy and delivery were uneventful. A family history revealed no other cases of neuromuscular diseases. The study was approved by the Ethics Committee of Sun Yat-sen Memorial Hospital of Sun Yat-sen University. Informed consent was obtained from the patients’ family.

When the patient was first observed in another hospital, the serum CK level was 3,008 mU/ml and the electroencephalogram (EEG) and survival of motor neuron 1 (SMN1) gene expression were normal. MRI revealed diffuse white matter dysplasia and the suspected diagnosis was adrenoleukodystrophy.

On physical examination, the patient’s chest and abdomen were normal, as were the results of cardiac assessment. However, the patient exhibited severe axial hypotonia with non-progressive bilateral upper and lower extremity weakness, which was more proximal than distal and predominantly at the shoulder and pelvic girdle. The muscle strength of the lower limb muscles was manually assessed using the standard Medical Research Council (MRC) scale (4), with a result of grade 3/5. The upper limb proximal muscle strength was determined to be grade 4/5. The cranial nerves were normal and the patient had no difficulties on sensory examination and coordination. Deep tendon reflexes and Babinski’s sign were negative. Tactile, pinprick and vibration sensations were normal.

Laboratory test results were as follows: leukocyte count, 8,900 cells/*μ*l; hemoglobin, 11.5 g/dl; hematocrit, 33.5%; and platelet count, 294×10^3^ cells/*μ*l. Biochemistry test results were as follows: glutamic oxaloacetic transaminase, 10 IU/l; glutamate alanine aminotransferase, 39 IU/l; CK, 1,556 IU/l; CK-MB, 98 IU/l; triglycerides, 0.88 mmol/l; and ammonia, 10.0 *μ*mol/l. Urine and blood screens for hereditary metabolic diseases were unremarkable.

### Neuroimaging

**Figure 1.**
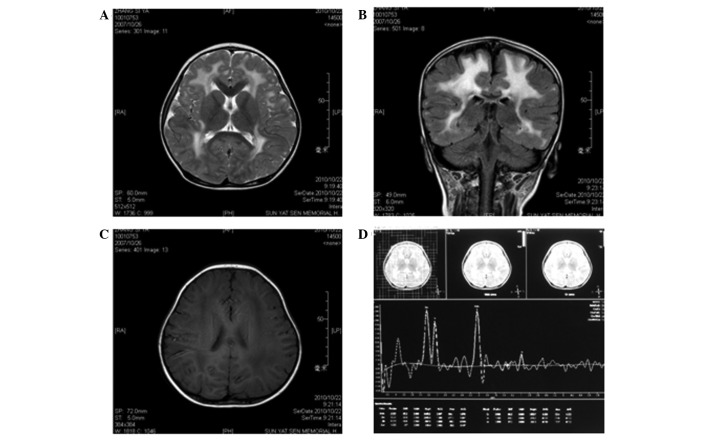
Magnetic resonance imaging (MRI) results at 3 years of age. (A) Axial T2-weighted image and (B) coronal T2-weighted fluid attenuated inversion recovery (FLAIR) image show diffuse, symmetrical high signal intensities in the cerebral white matter. (C) Axial T2-weighted image shows diffuse low signal intensity in the cerebral white matter. (D) MRS [TR/TE = (2,000 m/sec)/(35 m/sec)] of the parietal white matter at 3 years of age demonstrated that the NAA/Cr and Cho/Cr ratios were normal, but the MI/Cr ratio was slightly increased. TR, time of repetition; TE, time of echo; Cho, choline; Cr, creatine; MI, myo-inositol; NAA, N-acetylaspartate, MRS, magnetic resonance spectroscopy.

MRI was performed at Sun Yat-sen Memorial Hospital of Sun Yat-sen University when the patient was 3 years old. T1-weighted images revealed symmetrical, low signal intensity in the white matter of the frontal, parietal, temporal and occipital lobes; however, the cortex was normal (Fig. 1C). T2-weighted images revealed lesions with abnormally high signal intensity in the white matter of the frontal, parietal, temporal and occipital lobes (Fig. 1A and B). The ventricles, cerebellum, basal ganglia and pons were normal. Semi-quantitative magnetic resonance spectroscopy (MRS) revealed that the N-acetylaspartate/creatine (NAA/Cr) and choline/creatine (Cho/Cr) metabolite ratios were within normal ranges. However, the myoinositol/creatine (mI/Cr) metabolite ratio was slightly increased (Fig. 1D).

### Electrophysiological studies

Electromyography (EMG) was used to aid in differentiating whether the patient’s deficits were myogenic or neurogenic. Needle EMG of the left and right tibialis anterior muscle suggested a myopathic process with reduced recruitment potential, decreased amplitude and duration of response, appearance of variable small amplitudes and short-duration polyphasic myogenic waves. Nerve conduction studies (NCS) were performed for the left tibial and left deep peroneal nerves using conventional methods. These revealed a normal motor nerve conduction velocity (MCV) as follows: tibial posterior, 52 m/sec and deep peroneal, 50 m/sec (normal range, 50–58 m/sec in the legs).

### Molecular genetic testing

The patient in the present case was suspected of having MDC1A based on congenital hypotonia, delayed motor milestones and brain white matter abnormalities on MRI. Thus, molecular genetic testing was performed without a muscle biopsy. Genomic DNA from the patient and the patient’s parents was extracted from peripheral blood leukocytes using standard procedures. PCR and DNA direct sequencing were used to analyze all 65 exons of LAMA2 to determine if there were any gene mutations.

DNA analysis revealed that the patient had a homozygous nonsense mutation in the LAMA2 gene in exon 50; a C>T exchange in nucleotide 7147 causing a stop codon (Arg2383X stop). The patient’s parents were heterozygotes for this mutation. This finding confirmed the diagnosis of MDC1A.

## Discussion

MDC1A is caused by mutations in the LAMA2 gene and was first described by Tomé *et al* in 1994 (2). The estimated prevalence of CMDs is ~1 in 7 million (5). In Europe, MDC1A accounts for ~40% of CMD cases (6). MDC1A is characterized by congenital muscle hypotonia, delayed or arrested motor milestones and feeding difficulties. Muscle weakness is absent or slowly progressive and is accompanied by contractures that mostly affect the elbows, hips, knees and ankles. The majority of patients may achieve unsupported sitting; however, <10% achieve ambulation (7). The common life-threatening complications of MDC1A include respiratory failure and feeding difficulties.

The patient in the present study was only 4 years old and had not suffered from any severe respiratory infections. However, with this disease, pulmonary infection is the most common cause of mortality, which may occur during the first decade or anytime thereafter. Treatment with non-invasive ventilation and tracheostomy may greatly improve health.

In CMDs, the serum levels of CK are mildly to markedly elevated. In general, CMD subtypes with primary or secondary merosin deficiency, including dystroglycanopathies, show high serum CK concentrations, while those with no merosin deficiency show normal or mildly increased serum CK concentrations (8). In the present case, the serum CK level was elevated to 1,556 IU/l, which indicated primary or secondary merosin deficiency.

EMG and NCS are recommended for all patients with suspected CMDs to confirm myopathy and to exclude other diseases. In the present case, EMG confirmed a myopathic process with early recruitment and decreased amplitude and duration of response, while the results of NCS were normal. In a number of cases of MDC1A, mild neuropathic changes may be observed since laminin α-2 is absent in the basement membranes surrounding Schwann cells and myelin sheaths (9).

The majority of patients with MDC1A have normal intellectual and speech development, although cases of learning disabilities and mental retardation have been reported (10). Epilepsy has been estimated to occur in ~6–8% of these cases; seizures are partial and complex, with no consistent pattern (10). In the present case, an EEG was normal; however, it is essential that a standard EEG is performed periodically for MDC1A patients.

Despite a minority that has clinical central nervous system findings, a consistent finding common to all patients >6 months of age is the presence of cerebral white matter abnormalities on neuroimaging. In the present case, cranial MRI revealed signal abnormalities in the white matter of the frontal, parietal, temporal and occipital lobes, whereas the cortex was normal. Children may initially be misdiagnosed as having a leukodystrophy. White matter changes do not regress with time. Although the pathophysiology of the white matter changes has not been completely elucidated, the majority of investigators postulate that disruption of the blood-brain barrier associated with laminin α-2 leads to increased water content, which results in abnormal white matter signal intensity (11,12). The pattern of white matter abnormalities associated with MDC1A is characteristic as compared with other CMD subtypes. A small number of patients have structural changes with mild ventricular enlargement, focal cortical dysplasia, occipital polymicrogyria and hypoplasia of the pons and cerebellum (13).

Previously, a diagnosis of MDC1A was based on the clinical findings of severe congenital hypotonia, weakness associated with high CK blood levels, white matter abnormalities and dystrophy associated with negative immunostaining of biopsied muscle for merosin (14). A muscle biopsy appears to be an essential factor in the diagnosis of MDC1A. However, with the more widespread use of molecular genetic testing for confirming the diagnosis of a CMD subtype, the recent trend has been to perform molecular genetic testing without a muscle biopsy when the medical history, physical examination and neurological examination support the diagnosis of a CMD.

In the present case, due to the congenital hypotonia, delayed motor milestones, markedly elevated CK concentration and brain white matter abnormalities on MRI, the patient was suspected of having MDC1A. Thus, we directly proceeded to molecular genetic testing without performing a muscle biopsy. Ultimately, a nonsense mutation in the LAMA2 gene confirmed our diagnosis of MDC1A.

LAMA2 is located on chromosome 6q22–23 in humans and on chromosome 10 in mice (15). This gene comprises 65 exons that encode for the α2 chain subunit of laminin-2. Laminin-2 is a heterotrimer consisting of laminin α-2, β-1 and γ-1 subunits (16). Mutations in LAMA2 include nonsense, missense, deletion and splice-site mutations, which all result in a primary deficiency in the laminin α-2 chain of merosin (15,17). Thus, for MDC1A, an evaluation may proceed directly to molecular genetic testing without a biopsy, depending on a typical presentation and following exclusion of other more common diagnoses. By contrast, if multiple genes need to be tested, such as for confirming a diagnosis of a dystroglycanopathy, the immunohistochemical analysis of a muscle biopsy may identify the subtype prior to molecular genetic testing.

MDC1A is the most common form of CMD. MDC1A is caused by a mutation of LAMA2 located on human chromosome 6q22–23. The typical presentations of MDC1A are severe congenital hypotonia, muscle weakness, elevated serum levels of CK and white matter abnormalities. To confirm a diagnosis of MDC1A, the evaluation may proceed directly to LAMA2 molecular genetic testing without the need for a muscle biopsy.

## Abbreviations:

CMDs: congenital muscular dystrophies;
MDC1A: merosin-deficient congenital muscular dystrophy type 1A;
MRI: magnetic resonance imaging;
EEG: electroencephalogram;
SMN1: survival of motor neuron 1;
MRC: Medical Research Council;
CK: creatine kinase;
CK-MB: creatine kinase-MB;
MRS: magnetic resonance spectroscopy;
NAA/Cr: N-acetylaspartate/creatine;
Cho/Cr: choline/creatine;
MI/Cr: myoinositol/creatine;
EMG: electromyography;
NCS: nerve conduction studies;
MCV: motor nerve conduction velocity;
LAMA2: laminin α-2 gene
